# Effects of antidiabetic drugs on left ventricular function/dysfunction: a systematic review and network meta-analysis

**DOI:** 10.1186/s12933-020-0987-x

**Published:** 2020-01-22

**Authors:** Da-Peng Zhang, Li Xu, Le-Feng Wang, Hong-Jiang Wang, Feng Jiang

**Affiliations:** Heart Center and Beijing Key Laboratory of Hypertension, Beijing Chaoyang Hospital, Capital Medical University, No. 8, Gongti South Road, Chaoyang District, Beijing, 100020 China

**Keywords:** GLP-1 agonists, SGLT-2 inhibitors, DPP-4 inhibitors, Left ventricular remodeling, LVEF

## Abstract

**Background:**

Although a variety of antidiabetic drugs have significant protective action on the cardiovascular system, it is still unclear which antidiabetic drugs can improve ventricular remodeling and fundamentally delay the process of heart failure. The purpose of this network meta-analysis is to compare the efficacy of sodium glucose cotransporter type 2 (SGLT-2) inhibitors, dipeptidyl peptidase-4 (DPP-4) inhibitors, glucagon-like peptide-1 (GLP-1) agonists, metformin (MET), sulfonylurea (SU) and thiazolidinediones (TZDs) in improving left ventricular (LV) remodeling in patients with type 2 diabetes (T2DM) and/or cardiovascular disease (CVD).

**Methods:**

We searched articles published before October 18, 2019, regardless of language or data, in 4 electronic databases: PubMed, EMBASE, Cochrane Library and Web of Science. We included randomized controlled trials in this network meta-analysis, as well as a small number of cohort studies. The differences in the mean changes in left ventricular echocardiographic parameters between the treatment group and control group were evaluated.

**Results:**

The difference in the mean change in LV ejection fraction (LVEF) between GLP-1 agonists and placebo in treatment effect was greater than zero (MD = 2.04% [0.64%, 3.43%]); similar results were observed for the difference in the mean change in LV end-diastolic diameter (LVEDD) between SGLT-2 inhibitors and placebo (MD = − 3.3 mm [5.31, − 5.29]), the difference in the mean change in LV end-systolic volume (LVESV) between GLP-1 agonists and placebo (MD = − 4.39 ml [− 8.09, − 0.7]); the difference in the mean change in E/e′ between GLP-1 agonists and placebo (MD = − 1.05[− 1.78, − 0.32]); and the difference in the mean change in E/e′ between SGLT-2 inhibitors and placebo (MD = − 1.91[− 3.39, − 0.43]).

**Conclusions:**

GLP-1 agonists are more significantly associated with improved LVEF, LVESV and E/e′, SGLT-2 inhibitors are more significantly associated with improved LVEDD and E/e′, and DPP-4 inhibitors are more strongly associated with a negative impact on LV end-diastolic volume (LVEDV) than are placebos. SGLT-2 inhibitors are superior to other drugs in pairwise comparisons.

## Background

In recent years, many studies have found that a variety of antidiabetic drugs exert significant protective action on the cardiovascular system, a mechanism that may be partially independent of hypoglycemic effects. This undoubtedly sounds like exciting news for diabetic patients, especially those with cardiovascular disease, although the cardiovascular protective mechanism is not clear. The sodium glucose cotransporter type 2 (SGLT-2) inhibitor empagliflozin has been demonstrated to reduce cardiovascular mortality by 38% and heart failure (HF) hospitalizations by 35% in patients with type 2 diabetes (T2DM) in the EMPA-REG OUTCOMES clinical trial [1, 2]. An early nonrandomized pilot study showed improved left ventricular function when glucagon-like peptide-1 (GLP-1) agonists were infused in patients with acute myocardial infarction and HF [3]. Two small randomized controlled trials showed that GLP-1 agonist infusion exerted a positive effect on patients with ischemic heart disease [4, 5]. Traditional hypoglycemic drugs such as metformin (MET) have also been found to have a positive effect on cardiovascular protection [6–8]. A 2019 meta-analysis found that MET reduced cardiovascular mortality and the incidence of cardiovascular events in diabetic patients [9]. These results suggest that we should prioritize drugs that have some cardiovascular protective effects when choosing a treatment for diabetes.

The mitigation of left ventricular (LV) remodeling was paralleled by improvements in LV systolic performance. If ventricular remodeling can be delayed, the process of HF can be fundamentally delayed. Recently, a study found that empagliflozin-treated pigs showed higher LV ejection fraction (LVEF) and significantly greater contractile reserve than control animals [10]. Empagliflozin improved adverse anatomic LV remodeling, enhanced left ventricular systolic function, and inhibited neurohormonal activation. Liraglutide [11], a GLP-1 agonist, slightly increased LVEF in patients with ST-segment elevation myocardial ischemia (STEMI) who underwent direct percutaneous coronary intervention. Alogliptin, a DPP-4 inhibitor, improves coronary flow reserve (CFR) and LVEF in patients with T2DM with coronary artery disease (CAD), and the improvement in CFR was associated with increased LV systolic function [12]. Ventricular remodeling is an important determinant of patient morbidity and long-term prognosis [13]. Although many antidiabetic drugs have the effect of reducing cardiovascular death and adverse cardiovascular events, it is still unclear which antidiabetic drugs can improve ventricular remodeling and fundamentally delay the process of HF. If drugs can be found to improve ventricular remodeling, it will be of great significance for patients with T2DM with cardiovascular disease (CVD).

The purpose of this network meta-analysis is to compare the efficacy of SGLT-2 inhibitors, DPP-4 inhibitors, GLP-1 agonists, MET, sulfonylurea (SU) and thiazolidinediones (TZDs) in improving LV remodeling in patients with T2DM and/or CVD.

## Methods

### Protocol and guidance

This systematic review and network meta-analysis followed the Preferred Reporting Items for Systematic Reviews and Meta analyses (PRISMA) guidelines [14].

### Eligibility criteria

We included randomized controlled trials with parallel group or crossover designs in this network meta-analysis, as well as a small number of cohort studies. All trials had to include treatment with one of the following 6 drugs or multiple drugs: MET, GLP-1 agonists, DPP-4 inhibitors, SGLT-2 inhibitors, TZDs and SU. The control group was treated with placebo or one of the 6 drugs. Study participants were either type 2 diabetic patients with or without CVD or patients with CVD alone. The outcome of the included studies must contain at least one of the following 6 cardiac function and structure measures: LVEF, LV end-diastolic diameter (LVEDD), LV end-systolic diameter (LVESD), LV end-diastolic volume (LVEDV), LV end-systolic volume (LVESV), LV mass index (LVMI), early diastolic velocity (e′), early diastolic to late diastolic velocities ratio (E/A) and mitral inflow E velocity to tissue Doppler e′ ratio (E/e′).

### Information sources and search strategy

We searched articles published before October 18, 2019, regardless of language or data, in 4 electronic databases: PubMed, EMBASE, Cochrane Library and Web of Science. The articles were selected by manual screening.

The following terms were used in the search: ventricular remodeling OR cardiac reverse remodeling OR CRR OR cardiac remodeling OR left ventricular remodeling OR left ventricular dysfunction OR LVD OR ejection fraction OR EF OR left ventricular ejection fraction OR LVEF OR end-diastolic volume OR EDV OR end-diastolic dimension OR EDD OR end-systolic volume OR ESV OR end-systolic dimension OR ESD OR LVEDD OR left ventricular end-diastolic dimension OR LVEDV OR left ventricular end-diastolic volume OR LVESD OR left ventricular end-systolic dimension OR LVESV OR left ventricular end-systolic volume OR left ventricular diameter OR left ventricular volume OR left ventricular mass index OR LVMI OR left atrial volume OR LAV OR left atrial volume index OR LAVI) AND (Dipeptidyl peptidase-4 inhibitors OR DPP-4 inhibitors OR Sodium-Glucose Cotransporter-2 Inhibitors OR SGLT-2 inhibitors OR Glucagon-like peptide-1 agonists OR GLP-1 agonists OR exenatide OR Lyxumia OR liraglutide OR Saxenda OR Tanzeum OR albiglutide OR Trulicity OR dulaglutide OR canagliflozin OR dapagliflozin OR empagliflozin OR ertugliflozin OR ipragliflozin OR luseogliflozin OR tofogliflozin OR sitagliptin OR vildagliptin OR saxagliptin OR alogliptin OR linagliptin OR gemigliptin OR teneligliptin OR metformin OR sulfonylureas OR glibenclamide OR glyburide OR glybenzcyclamide OR gliquidone OR Gliclazide OR glipizide OR Glucotrol OR gliclazide OR Gliclarizonaide OR glimepiride OR Amaryl OR acarbose OR Precose OR air conditioningarbose OR miglitol OR Glyset OR voglibose OR Basen OR repaglinide OR nateglinide OR mitiglinide.

### Study selection

Two methodologically trained independent reviewers screened titles and abstracts to determine whether they met the eligibility criteria. The reviewers read the full text and extracted relevant data after consensus was reached. Any differences were resolved through discussion and arbitration, if necessary, by a third reviewer. The reasons for inclusion or exclusion are recorded in detail. Case reports, letters and minutes of meetings were excluded. The PRISMA flow diagram was used to summarize the study selection processes.

### Data extraction

Two investigators used a predefined data extraction sheet to independently extract data from each included study, such as authors, publication year, study design, population, subject ages, intervention, male sex, sample size, grouping and number of people in the group, baseline and endpoint data, including counts and effect estimates (mean ± SD), country, follow-up months, title, and conclusion. The third investigator independently reviewed the data to ensure accuracy. If no data in digital format were available, we used the free software Plot Digitizer to estimate data from the graphs.

### Definition of outcomes

The outcome of this meta-analysis was the difference in the mean change in echocardiographic parameters between the treatment group and control group. The echocardiographic parameters included LVEF, LVEDD, LVESD, LVEDV, LVESV, LVMI, e′, E/A and E/e′.

### Statistical analysis

We used the network meta-analysis approach to evaluate the comparative effect by combining direct and indirect evidence of all relevant treatment effects. To visualize network geometry and node connectivity, we summarized the geometry of the evidence network using network plots. We conducted a network meta-analysis of the comparative efficacy using a multivariate random-effects (restricted maximum likelihood estimation) meta-analysis model. For all treatment comparisons, we present summary mean differences and 95% confidence intervals. To obtain treatment hierarchies, we used a parametric bootstrap procedure with 5000 resamples to compute ranking probabilities. Mean rankings as well as surface under the cumulative ranking curve (SUCRA) values were computed for each treatment. We checked the consistency of the network using local and global inconsistency tests. The local inconsistency test evaluates the loop inconsistency of all the triangle loops on the network. Global inconsistency is a goodness-of-fit test. If any relevant sources of bias were found, we performed sensitivity analyses. All analyses were conducted in Stata/SE, version 14.

### Assessment of risk of bias in individual studies

Each study was evaluated using the Cochrane tool. Potential sources of bias include random sequence generation, allocation concealment, blinding of participants and staff, blinding of outcome assessors, incomplete outcome data, and selective reporting. Each trial received a study level score of low, high, or unclear risk of bias for each domain. Two authors independently conducted this assessment, and discrepancies were resolved by consensus.

### Assessment of small study effects

To evaluate the presence of small study effects, we visually inspected comparison-adjusted funnel plots for each outcome. We produced funnel plots for all comparisons concerning the difference in LVEF change between treatment and placebo.

## Results

### Study selection

**Fig. 1:**
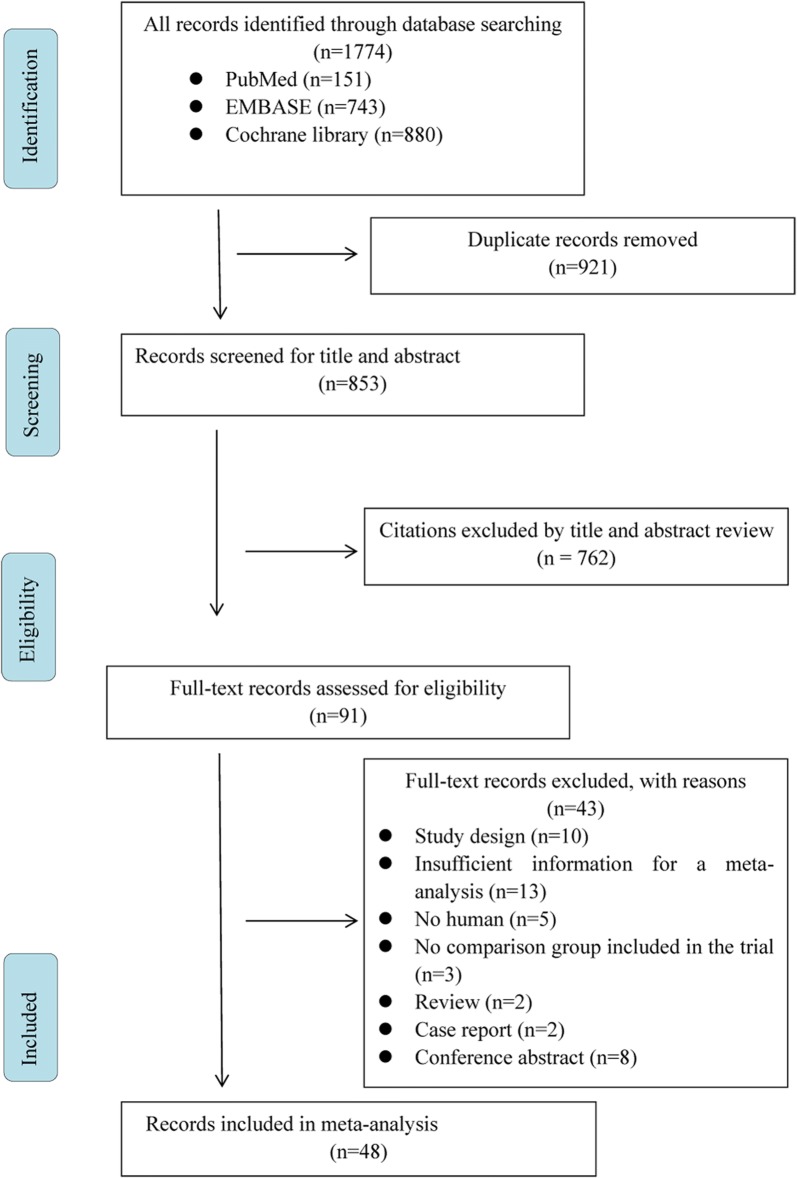
Flowchart of study selection

The initial search of 4 databases yielded 1774 articles. We obtained 91 articles after reading the title and abstract, excluding duplicates and irrelevant articles. After screening the full texts manually, 43 articles were excluded for reasons including study design (n = 10), insufficient information for a meta-analysis (n = 13), no human subjects (n = 5), no comparison group included in the trial (n = 3), review article (n = 2), case report (n = 2), and conference abstract (n = 8). Eventually, 48 studies were included in this network meta-analysis (Fig. 1).

### Study characteristics

**Fig. 2:**
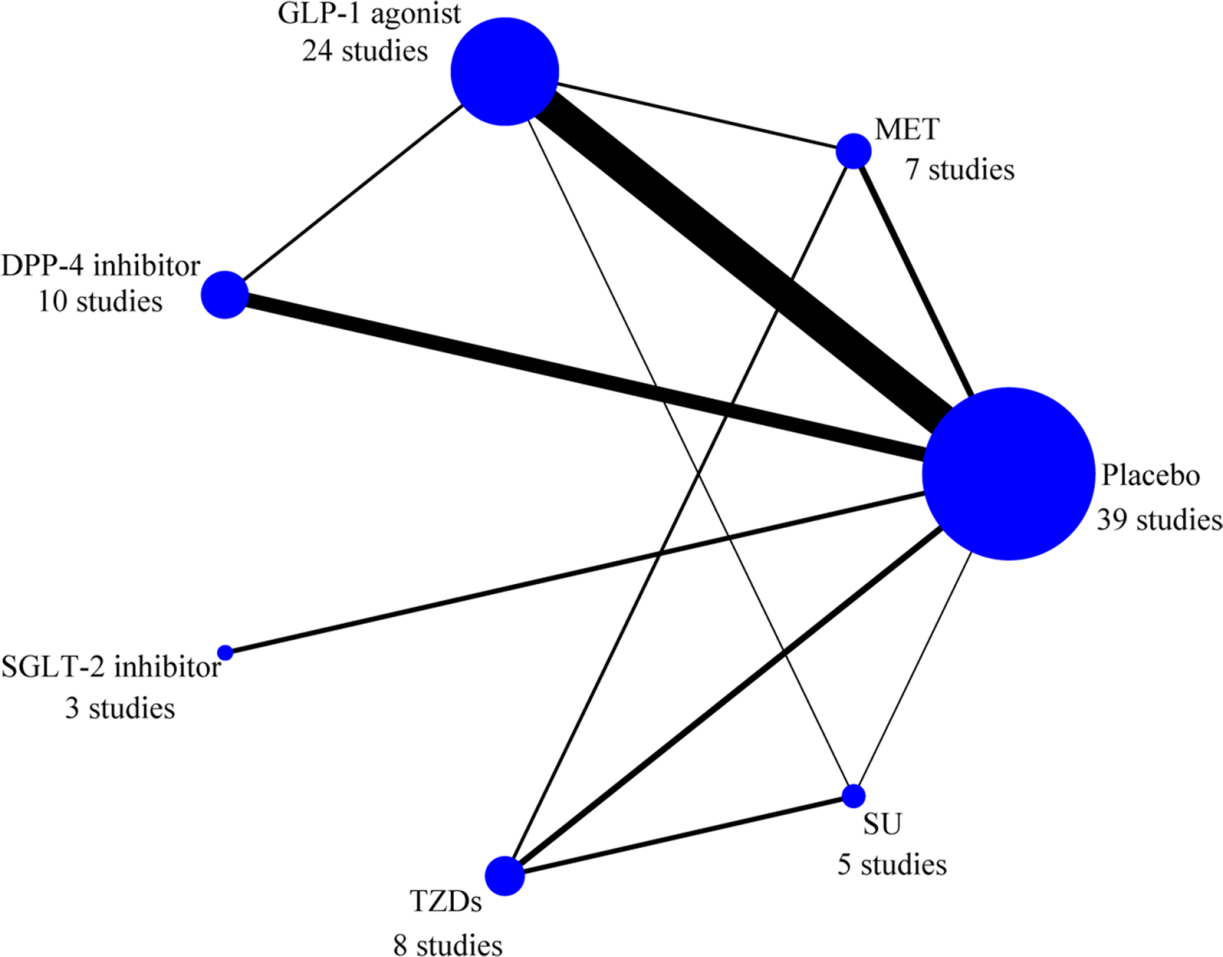
Network plot for all studies

In this network meta-analysis, 48 studies were included, comprising a total sample size of 4790 participants. The 48 studies included 36 randomized controlled trials (RCTs) and 12 cohort studies. Among them, 7 trials concerned MET, 25 trials involved GLP-1 agonists, 10 studies reported DPP-4 inhibitors, 3 studies discussed SGLT-2 inhibitors, 8 studies referred to TZDs and 5 studies covered SU. Among them, 39 studies reported pairwise comparisons with placebo, 2 were three-arm studies (placebo was one arm), and 7 were comparisons between two treatments. Participants in 17 trials were patients with T2DM, participants in 9 studies were patients with CVD, and participants in the remaining 22 studies were patients with T2DM and CVD. The summary data of each included study are shown in Table 1, and the network plot is shown in Fig. 2.

**Table 1:** Characteristics of included studies

Study	Year	Age	Male%	Patients	Sample size	Treatment	Country
Al Ali et al. [15]	2016	57.9 (11.4)	80.5	CVD	237	MET	Netherlands
Arturi et al. [16]	2017	59.5 (9)	70.0	T2DM	32	GLP-1 agonist, DPP-4 inhibitor	Italy
Bizino et al. [17]	2019	60 (6)	61.0	T2DM	49	GLP-1 agonist	Netherlands
Bonora et al. [18]	2019	65.7 (5.9)	66.7	T2DM	30	SGLT-2 inhibitor	Italy
Brenne et al. [19]	2016	61.3 (11.0)	79.3	CVD	173	DPP-4 inhibitor	Austria
Chen et al. [20]	2017	66 (5)	100.0	T2DM + CVD	23	GLP-1 agonist	Netherlands
Chen et al. [21]	2016	58.0 (11.7)	76.0	T2DM + CVD	90	GLP-1 agonist	China
Chen et al. [22]	2015	57.7 (11.3)	67.0	T2DM + CVD	92	GLP-1 agonist	China
Cohen et al. [23]	2019	62.9 (6.8)	58.8	T2DM	25	SGLT-2 inhibitor	Australia
Ghazzi et al. [24]	1997	54 (10.8)	54.0	T2DM	154	TZDs, SU	USA
Giles et al. [25]	2008	64.2 (9.92)	70.2	T2DM + CVD	518	TZDs, SU	USA
Halbirk et al. [26]	2010	61 (3)	86.7	CVD	15	GLP-1 agonist	Denmark
Hiramatsu et al. [27]	2018	70.5 (5.7)	–	T2DM + CVD	98	GLP-1 agonist, DPP-4 inhibitor	Japan
Hiramatsu et al. [28]	2015	68.5 (9.4)	86.7	T2DM	30	GLP-1 agonist	Japan
Jorgensen et al. [29]	2017	57 (10)		T2DM	32	GLP-1 agonist	Denmark
Jorsal et al. [30]	2017	65 (9.2)	89.3	T2DM + CVD	241	GLP-1 agonist	Denmark
Kato et al. [12]	2016	73.3 (6.6)	60.0	T2DM + CVD	20	DPP-4 inhibitor	Japan
Kumarathurai et al. [31]	2016	61.8 (7.6)	79.0	T2DM + CVD	39	GLP-1 agonist	Denmark
Lambadiari et al. [32]	2018	51 (12)	66.7	T2DM	60	GLP-1 agonist, MET	Greece
Lepore et al. [33]	2016	58 (10)	74.0	CVD	56	GLP-1 agonist	USA
Leung et al. [34]	2016	56 (6)	56.0	T2DM	75	DPP-4 inhibitor	Australia
Lips et al. [35]	2017	–	–	T2DM + CVD	38	GLP-1 agonist	Czech Republic
Liu et al. [36]	2017	58 (15)	53.3	T2DM	120	GLP-1 agonist, MET	China
Margulies et al. [37]	2016	62 (52–68)	80.0	T2DM + CVD	300	GLP-1 agonist	USA
Mcmurray et al. [38]	2018	62.9 (8.5)	77.3	T2DM + CVD	254	DPP-4 inhibitor	UK
Mohan et al. [39]	2019	64.5 (8.9)	84.0	CVD	63	MET	UK
Naka et al. [40]	2010	64.3 (8.1)	36.0	T2DM + CVD	81	TZDs	Greece
Nielsen et al. [41]	2019	66 (7)	94.4	CVD	36	GLP-1 agonist	Denmark
Nikolaidis et al. [42]	2004	58 (3)	70.0	T2DM + CVD	21	GLP-1 agonist	USA
Nogueira et al. [43]	2014	57 (7)	50	T2DM	29	DPP-4 inhibitor	Brazil
Nozue et al. [44]	2016	68 (10)	100.0	T2DM + CVD	15	GLP-1 agonist	Japan
Nystrom et al. [45]	2017	61 (7.6)	72.7	T2DM + CVD	62	GLP-1 agonist, SU	Sweden
Oe et al. [46]	2015	67.8 (10.5)	50.0	T2DM + CVD	80	DPP-4 inhibitor	Japan
Otagaki et al. [47]	2019	70 (54–72)	71.0	T2DM	42	SGLT-2 inhibitor	Japan
Ozawa et al. [48]	2009	67.6 (8.8)	75.0	T2DM + CVD	54	TZD	Japan
Sardu et al. [49]	2018	72 (7)	71.5	T2DM + CVD	559	GLP-1 agonist	Italy
Scognamiglio et al. [50]	2002	61 (7)	73.7	T2DM + CVD	38	SU	Italy
Sokos et al. [51]	2006	61 (4)	58.3	T2DM + CVD	21	GLP-1 agonist	USA
St John Sutton et al. [52]	2002	56.1 (8.9)	71.0	T2DM	203	TZDs, SU	USA
Türkmen Kemal et al. [53]	2007	55.92 (8.26)	23.1	T2DM	46	TZD, MET	Turkey
Van Der Meer et al. [54]	2009	56.8 (1.0)	–	T2DM	78	TZD, MET	Netherlands
Wägner et al. [10]	2019	53.2 (9.7)	41.7	T2DM	24	GLP-1 agonist	Spain
Wong et al. [55]	2012	64 (8)	90.0	CVD	61	MET	UK
Woo et al. [56]	2013	59.5 (13.2)	89.0	CVD	58	GLP-1 agonist	Korea
Yamada et al. [57]	2017	69 (8)	69.1	T2DM	115	DPP-4 inhibitor	Japan
Yamamoto et al. [58]	2017	71 (10)	62.0	T2DM + CVD	158	DPP-4 inhibitor	Japan
Yokoyama et al. [59]	2007	63 (10)	84.0	T2DM + CVD	93	TZD	Japan
Zhang et al. [60]	2017	59.1 (11.8)	77.0	CVD	52	GLP-1 agonist	China
Total studies [48]					4790		

*CVD* cardiovascular disease, *DPP-4* dipeptidyl peptidase-4; *GLP-1* glucagon-like peptide-1, *MET* metformin, *SGLT-2* sodium glucose cotransporter type 2, *SU* sulfonylurea, *T2DM* type 2 diabetes mellitus, *TZDs* thiazolidinediones

### Risk of bias within studies

Among the 10 cohort studies, high risk was observed in randomization and blinding. Among the 36 RCTs, high risk was observed in the blinding of participants and staff, as 11 were open-label, but most of their blinding of outcome assessors was at low risk. No risk of incomplete outcome data or selective reporting was identified in any study (Additional file 1: Figure S1 and Additional file 2: Figure S2).

### Synthesis of results

#### Difference in mean change in LVEF

**Fig. 3:**
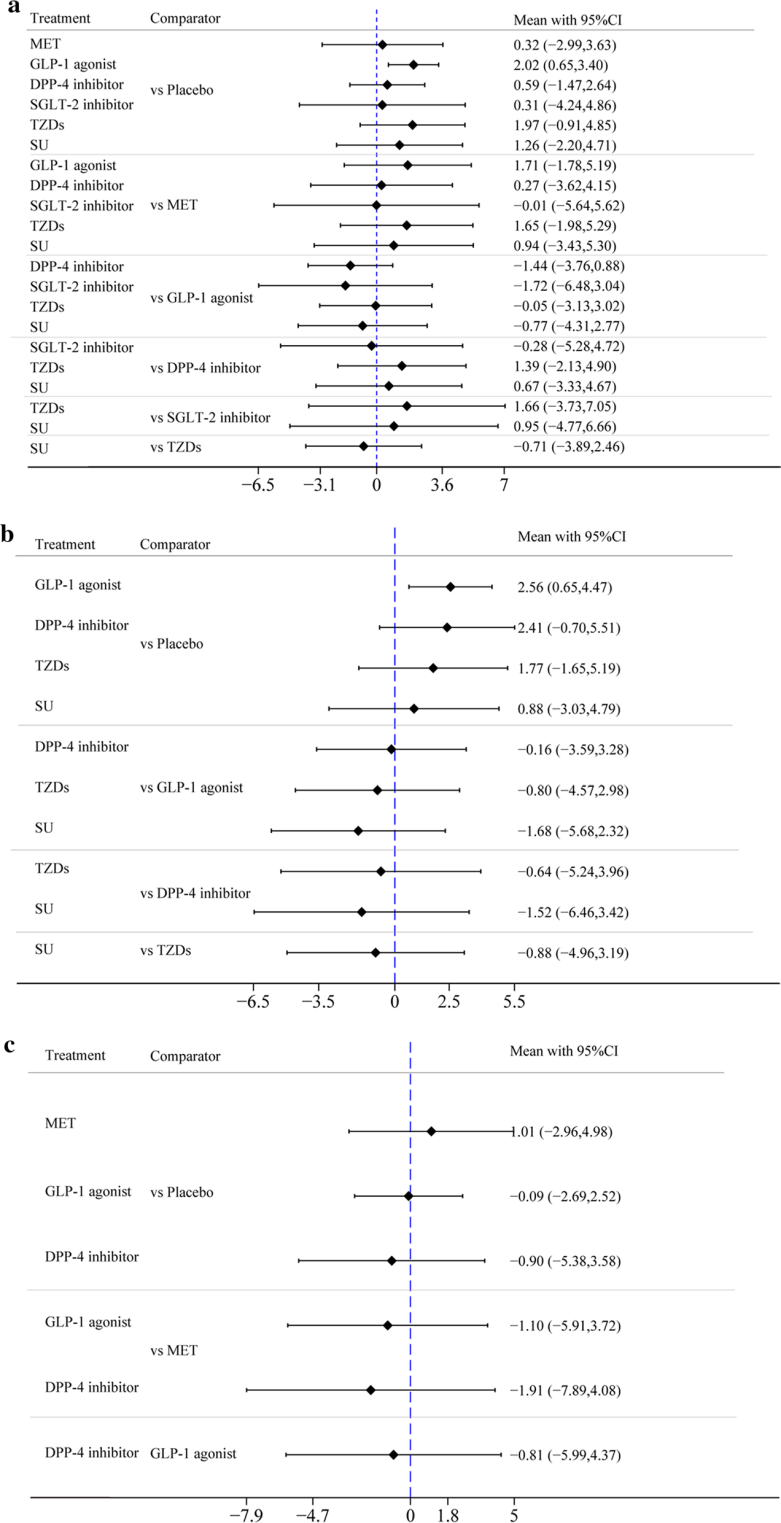
**a** Forest plot of mean difference of LVEF%. **b** Forest plot of mean difference of LVEF% among patients with T2DM + CVD. **c** Forest plot of mean difference of LVEF% among patients with CVD without T2DM

First, the difference in the mean change in LVEF between GLP-1 agonists and placebo in treatment effect was greater than zero (mean difference (MD) = 2.04% [95% confidence interval (CI) 0.64%, 3.43%]), indicating that GLP-1 agonists were more significantly associated with improved LVEF than placebo. Second, there was no difference in the mean change in LVEF between any of the other 5 drugs (i.e., MET, DPP-4 inhibitors, SGLT-2 inhibitors, TZDs, and SU) and placebo in treatment effect, as well as no difference in treatment effect in the pairwise comparison between any two of the 6 drugs (Fig. 3a, Table 2a).

**Table 2:** Studies included in comparisons. (a) 1 LVEF 44 trails, (b) LVEDD 8 trails, (c) LVESD 6 trails, (d) LVEDV 17 trails, (e) LVESV 15 trails, (f) LVMI 15 trails, (g) E/e′ 11 trails, (h) e′ 5 trails, (i) E/A 14 trails included

Treatment	No. of trials	Total no. of patients
a) LVEF 44 trails		
Control	38	1635
DPP-4 inhibitor	9	421
GLP-1 agonist	24	991
MET	5	242
SGLT-2 inhibitor	3	53
SU	4	242
TZDs	7	357
b) LVEDD 8 trails		
Control	7	542
DPP-4 inhibitor	2	126
GLP-1 agonist	4	399
MET	1	30
SGLT-2 inhibitor	1	21
TZDs	1	42
c) LVESD 6 trails		
Control	6	476
DPP-4 inhibitor	1	55
GLP-1 agonist	3	369
SGLT-2 inhibitor	1	21
TZDs	1	42
d) LVEDV 17 trails		
Control	15	915
DPP-4 inhibitor	2	163
GLP-1 agonist	11	621
MET	2	157
SGLT-2 inhibitor	1	17
SU	1	60
TZDs	2	97
e) LVESV 15 trails		
Control	14	858
DPP-4 inhibitor	1	92
GLP-1 agonist	11	621
MET	3	173
TZDs	2	52
f) LVMI 15 trails		
Control	11	416
DPP-4 inhibitor	5	162
GLP-1 agonist	5	92
MET	2	149
SU	3	249
TZDs	4	243
g) E/e′ 11 trails		
Control	9	430
DPP-4 inhibitor	3	72
GLP-1 agonist	6	226
MET	1	118
SGLT-2 inhibitor	1	21
SU	1	22
TZDs	1	42
h) e′ 5 trails		
Control	5	341
DPP-4 inhibitor	2	40
GLP-1 agonist	1	122
MET	1	118
TZDs	1	42
i) E/A 14 trails		
Control	10	378
DPP-4 inhibitor	4	82
GLP-1 agonist	8	200
MET	4	217
SGLT-2 inhibitor	1	21
TZDs	2	81

*e′* early diastolic velocity, *E/e′* mitral inflow E velocity to tissue Doppler e′ ratio, *E/A* early diastolic to late diastolic velocities ratio, *DPP-4* dipeptidyl peptidase-4; *GLP-1* glucagon-like peptide-1, *MET* metformin, *SGLT-2* sodium glucose cotransporter type 2, *SU* sulfonylurea, *TZDs* thiazolidinediones

We performed 2 subgroup analyses of T2DM + CVD and CVD without T2DM and obtained the results as shown in Fig. 3b, c. No significant difference in LVEF improvement was demonstrated between GLP-1 agonists and placebo in the CVD without T2DM subgroup, with a difference in mean change of − 0.09 (− 2.69, 2.52). However, GLP-1 agonists were shown to be more significantly associated with LVEF improvement than placebo in the T2DM + CVD subgroup, in which the difference in mean change was 2.56 (0.65, 4.47). This finding showed that GLP-1 agonists had a better effect on diabetic patients with CVD than on patients with CVD alone. No significant difference in change in LVEF was demonstrated in the 2 subgroups between other drugs and placebo or in pairwise comparisons.

#### Difference in mean change in LVEDD

**Fig. 4:**
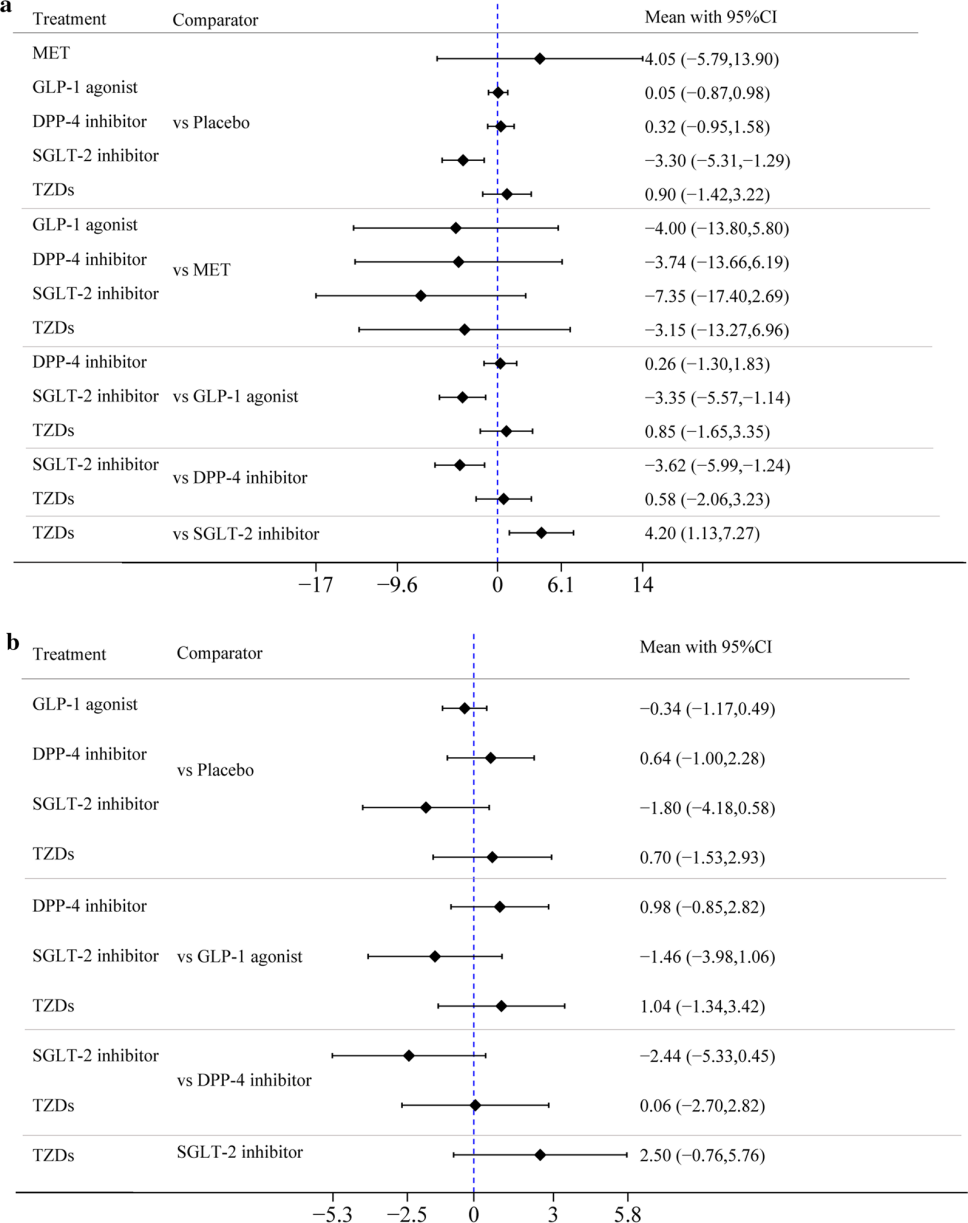
**a** Forest plot of mean difference of LVEDD. **b** Forest plot of mean difference of LVESD

First, the difference in the mean change in LVEDD between SGLT-2 inhibitors and placebo in treatment effect was less than zero (MD = − 3.3 mm [95% CI − 5.31, − 5.29], indicating that SGLT-2 inhibitors were more significantly associated with improved LVEDD than placebo. Second, there was no difference in the mean change in LVEDD between any of the other 5 drugs (MET, GLP-1 agonists, DPP-4 inhibitor, TZDs, and SU) and placebo in the treatment effect. Third, the MDs in pairwise comparisons between two drugs–STLG2 vs GLP-1 agonists, STLG2 vs DPP-4 inhibitors, and TZDs vs STLG2–in treatment effect were − 3.35 mm [95% CI − 5.57, − 1.14], − 3.62 mm [95% CI − 5.99, − 1.24], and 4.2 mm [95% CI 1.13, 7.27], respectively, showing that the improved effect of STLG2 on LVEDD was obviously better than those of GLP-1 agonists, DPP-4 inhibitors, and TZDs. There was no difference in the mean change in LVEDD before and after the use of any pair of other drugs in treatment effect (Fig. 4a, Table 2b).

#### Difference in mean change in LVESD

There was no difference in mean change in LVESD between each of the 6 drugs and placebo or in pairwise comparison between any two of the 6 drugs in treatment effect (Fig. 4b, Table 2c).

#### Difference in mean change in LVEDV

**Fig. 5:**
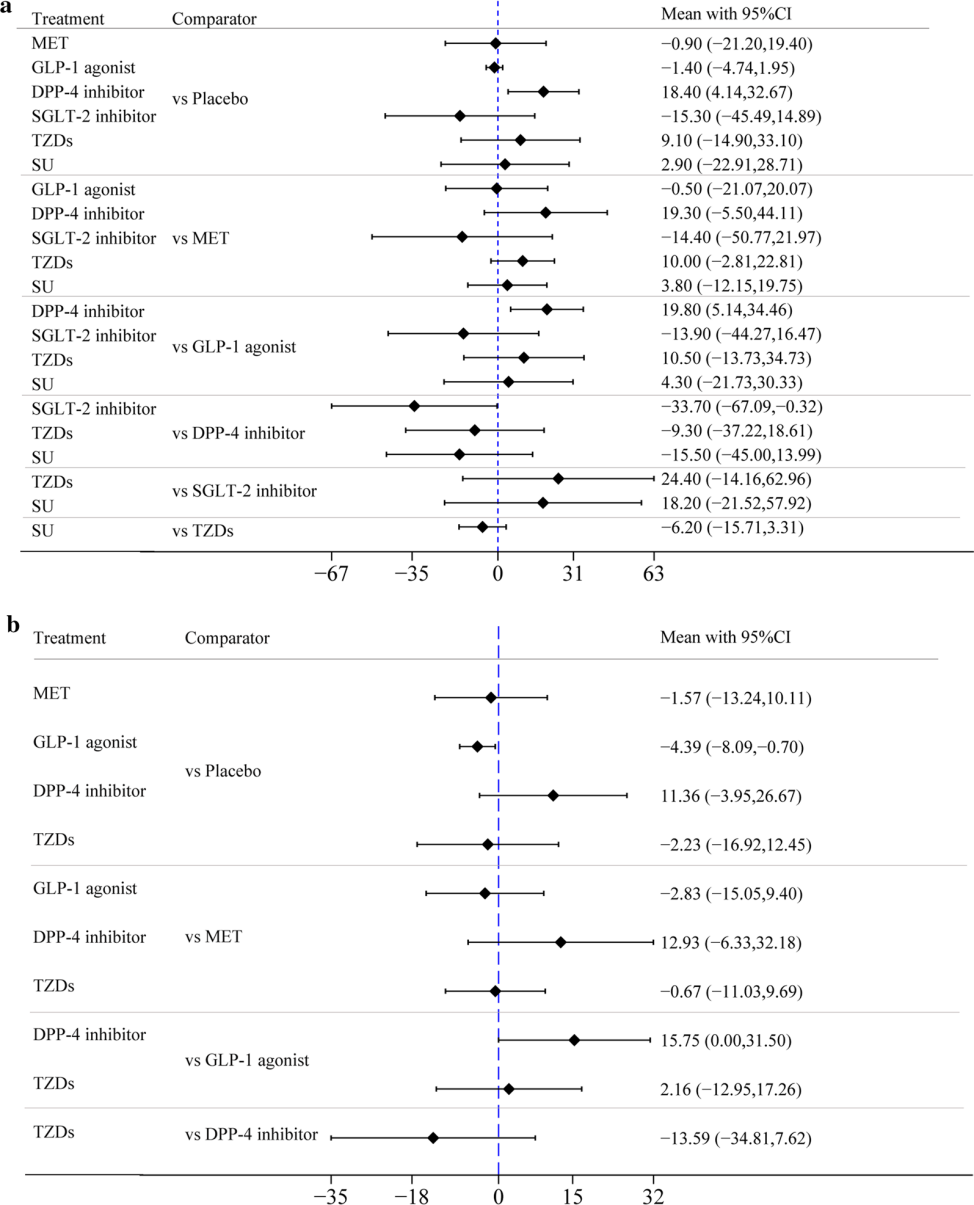
**a** Forest plot of mean difference of LVEDV. **b** Forest plot of mean difference of LVESV

First, the difference in the mean change in LVEDV between DPP-4 inhibitors and placebo in treatment effect was significantly larger than zero (MD = 18.4 ml [95% CI 4.14, 32.67]), indicating that DPP-4 inhibitors had a negative impact on LVEDV. Second, there was no difference in the mean change in LVEDV between any of the other 5 drugs (MET, GLP-1 agonists, SGLT-2 inhibitors, TZDs, and SU) and placebo, in treatment effect. Third, the MD between the two drugs–DPP-4 inhibitors vs GLP-1 agonists–in treatment effect was 19.8 ml [95% CI 5.14, 34.46], which was significant, indicating that DPP-4 inhibitors were more significantly associated with a negative impact on LVEDV than GLP-1 agonists. There was no difference in the mean change in LVEDV before and after the use of any pair of other drugs in treatment effect (Fig. 5a, Table 2d).

#### Difference in mean change in LVESV

First, the difference in the mean change in LVESV between GLP-1 agonists and placebo in treatment effect was less than zero (MD = − 4.39 ml [95% CI − 8.09, − 0.7]) with significance, indicating that GLP-1 agonists were more significantly associated with the improvement of LVESV than placebo. Second, there was no difference in the mean change in LVESV between any of the other 5 drugs (MET, SGLT-2 inhibitors, DPP-4 inhibitors, TZDs, and SU) and placebo, in the treatment effect. Third, the MD between the two drugs–DPP-4 inhibitors vs GLP-1 agonists–in treatment effect was 15.75 ml [95% CI 0.00. 31.5] with significance, showing that DPP-4 inhibitors were more significantly associated with a negative impact on LVESV than GLP-1 agonists. There was no difference in the mean change in LVESV before and after the use of any pair of other drugs in treatment effect (Fig. 5b, Table 2e).

#### Difference in mean change in LVMI

**Fig. 6:**
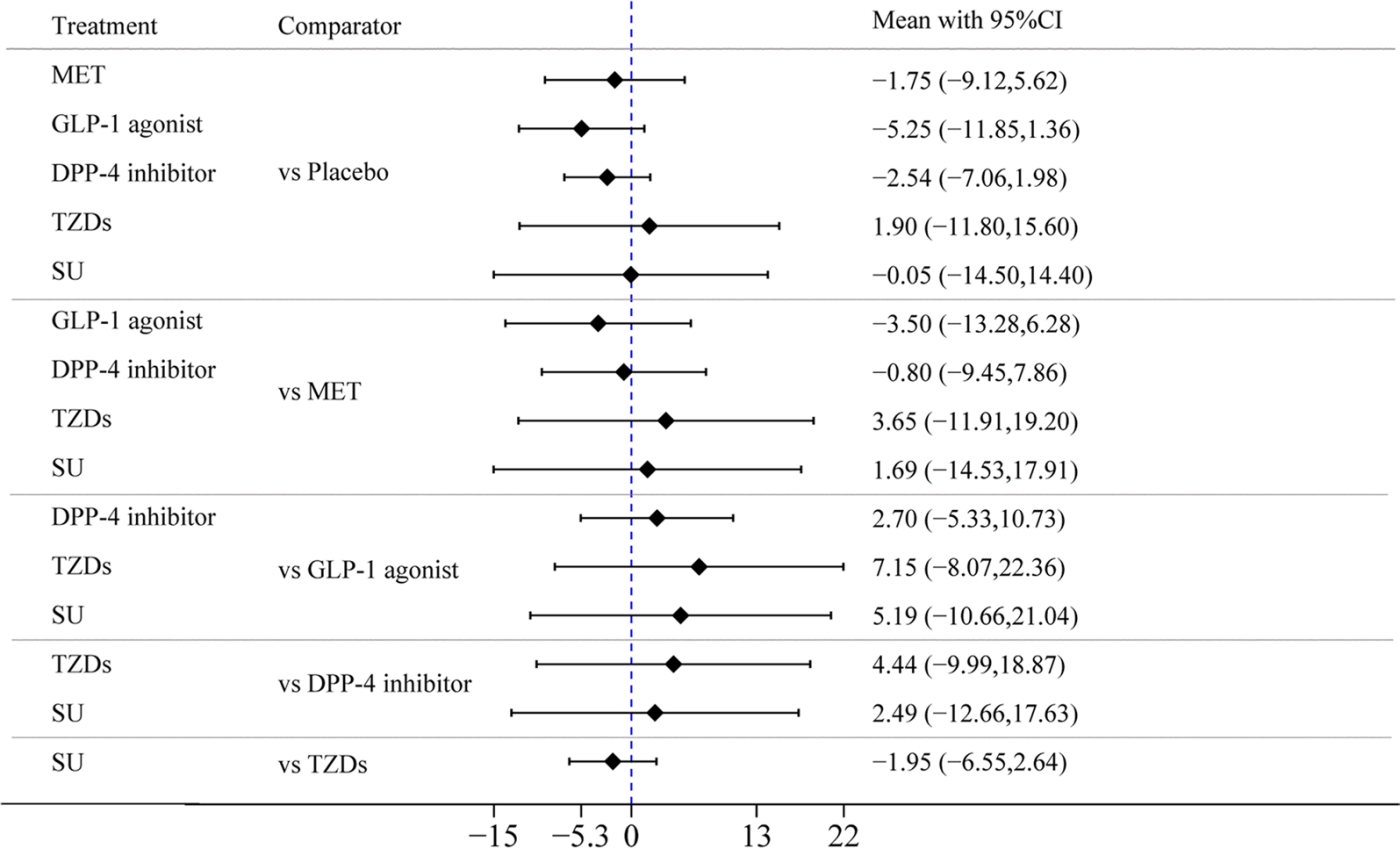
Forest plot of mean difference of LVMI

There was no difference in mean change in LVMI between each of the 6 drugs and placebo or in pairwise comparison between any two of the 6 drugs in treatment effect (Fig. 6, Table 2f).

We can make clear from the above discussion that compared with placebo, GLP-1 agonists may notably improve LVEF and LVESV, STLG2 may obviously improve LVEDD, and DPP-4 inhibitors exert a negative impact on LVEDV. SGLT-2 inhibitors are superior to other drugs in pairwise comparison.

#### Difference in mean change in e′, E/A and E/e′

**Fig. 7:**
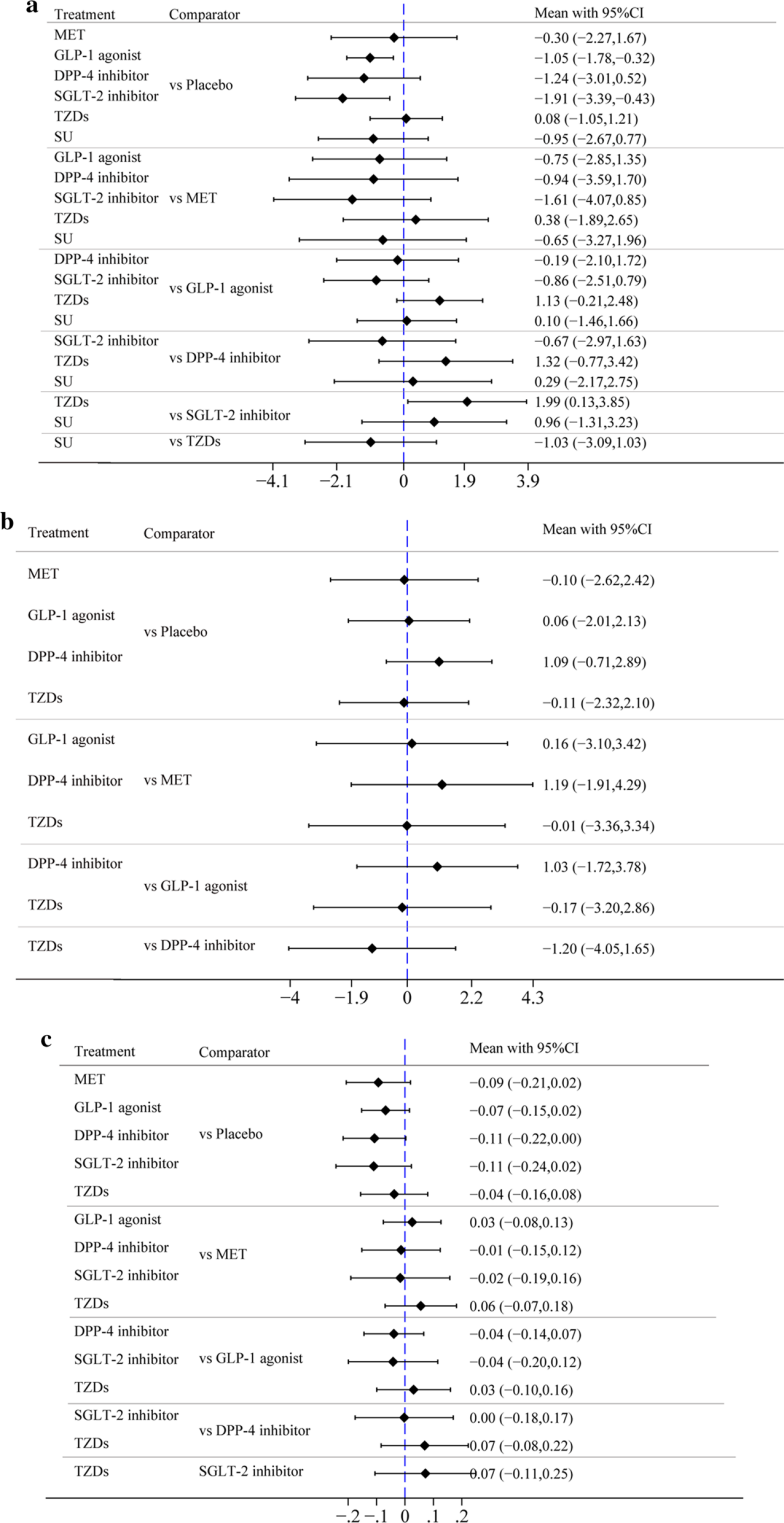
**a** Forest plot of mean difference of E/e.′ **b** Forest plot of mean difference of e′. **c** Forest plot of mean difference of E/A

GLP-1 agonists were more significantly associated with reducing E/e′ than was placebo, and the difference in mean change was − 1.05 (− 1.78, − 0.32). SGLT-2 inhibitors were more significantly associated with reducing E/e′ than was placebo, and the difference in mean change was − 1.91 (− 3.39, − 0.43). There was no significant difference in mean change in the treatment effect of e′ and E/A between any of the 6 drugs and placebo or in pairwise comparisons between any two of the 6 drugs (Fig. 7, Table 2g–i).

#### Ranking probabilities

According to the SUCRA results, the ranking of the efficacy of the 6 drugs and placebo is shown in Additional file 3: Table S1. GLP-1 agonists ranked first in the treatment effect on LVEF and LVMI, and SGLT-2 inhibitors ranked first in treatment effect on LVEDV, LVEDD, LVESD and E/e′. DPP-4 inhibitors ranked first in treatment effect on LVESV and e′.

#### Inconsistency test

No evidence for statistically significant inconsistency in any of the 6 echocardiographic parameters (global inconsistency tests P = 0.06 to 0.89) was found.

#### Risk of bias across studies

The funnel plots were made only for comparisons of differences in mean change in LVEF between the treatment and the placebo groups, as funnel plots are not feasible for those including fewer than 10 studies. Placebo vs. GLP-1 agonists (red A vs. C) and placebo vs. DPP-4 inhibitors (green A vs. D) were included in 24 and 9 studies, respectively, and no evidence of publication bias was found (Additional file 4: Figure S3).

## Discussion

In this network meta-analysis, 48 studies were included, comprising a total sample size of 4790 subjects. The 48 studies included 36 RCTs and 12 cohort studies. We found that compared with placebo, GLP-1 agonists increased LVEF and decreased LVESV and E/e′, and SGLT-2 inhibitors decreased LVEDD and E/e′. These results suggested that GLP-1 agonists and SGLT-2 inhibitors could improve ventricular remodeling.

Remodeling is an important determinant of patient morbidity and long-term outcomes. Adverse anatomical remodeling occurs at several levels, including anatomical, metabolic, and neurohormonal remodeling. Anatomical remodeling is characterized by LV dilatation, hypertrophy, and geometrical remodeling (the heart becomes more spherical).

Our meta-analysis revealed that GLP-1 agonist treatment triggered an increase in the mean change in LVEF% of 2.04% and a decrease in mean change in LVESV of 4.39 ml compared with placebo treatment. GLP-1 agonists were also demonstrated to significantly reduce E/e. Our subgroup analysis suggested that GLP-1 agonists had a better effect on diabetic patients with CVD than patients with CVD alone. GLP-1 agonists [61] are an incretin hormone secreted mainly by intestinal L-cells in response to the presence of nutrients. GLP-1 agonists mimic the effects of the native GLP-1 receptor, which increases insulin secretion, inhibits glucagon secretion, increases satiety and slows gastric emptying [33, 62]. However, the mechanism by which GLP-1 agonists exert their cardiovascular protective action, especially to improve ventricular remodeling, is still unclear. It was reported that liraglutide, one kind of GLP-1 agonist, inhibited angiotensin II and pressure overload-induced cardiac remodeling by regulating PI3K/Akt1 and AMPKα signaling [63]. Wang et al. [64] also found that the cardiac protection of GLP-1 agonists might be dependent on inhibition of oxidative stress through the mammalian target of rapamycin complex 1/p70 ribosomal protein S6 kinase pathway. In addition to their weight loss and glucose-lowering effects, GLP-1 agonists have been shown to protect the heart during acute ischemia and improve mitochondrial function, microvascular function, and myocardial glucose uptake in experimental animal models of heart failure [41, 65]. Giblett et al. [61] found that GLP-1 agonists are present in left ventricular cardiomyocytes but are not expressed in vascular tissue, so GLP-1 agonists may have direct ventricular effects and mediate secondary vasodilation through ventricular artery interactions. In addition, GLP-1 agonists increase natriuresis [17], reduce blood pressure, reduce inflammation, reduce ischemic injury, increase heart rate, increase plaque stabilization and decrease smooth muscle proliferation. It has been suggested that the positive effects of GLP-1 agonists on cardiovascular disease may be the result of a direct action on the arteriosclerotic process [66]. All these processes may leave an imprint on GLP-1 agonists’ role in the improvement of ventricular remodeling.

The results of our study revealed that SGLT-2 inhibitors could significantly reduce LVEDD but could not exert a significant effect on LVEDV. However, SGLT-2 inhibitors could significantly reduce E/e′ and improve diastolic function of the left ventricle. Some studies suggested that SGLT-2 inhibitors could reduce oxidative stress [66], thereby improving arteriosclerosis and endothelial dysfunction. Experimental data in obese and diabetic mice demonstrated that the SGLT-2 inhibitor empagliflozin significantly ameliorated cardiac fibrosis, coronary arterial thickening, and cardiac macrophage infiltration, suggesting a direct cardiac effect along with an attenuation of oxidative stress on the myocardium [67]. Previous studies have indicated that SGLT1 receptors are predominantly expressed in the human intestine, and the higher selectivity of SGLT1 receptors could lower the variations in postprandial blood glucose, which might help to reduce heart failure risk. These factors collectively could play a crucial role in reducing the vasculopathy burden on the heart. Other potential beneficial mechanisms, for instance, improved arterial compliance and so on, have also been postulated [67, 68]. Another study found that SGLT-2 inhibitors in addition to tofogliflozin administration had a favorable effect on left ventricular systolic and diastolic function in patients with T2DM [65, 69–71]. A network meta-analysis of 91 randomized trials by Yang et al. also found that in terms of heart failure risk, sodium-glucose cotransporters 2 were the most favorable option among all classes of antidiabetic medications [72]. Although SGLT-2 inhibitors seemed to reduce the risk of heart failure, Shao et al. considered that dapagliflozin might have greater effects on heart failure reduction compared to empagliflozin [73]. This study has shown the effect of SGLT-2 inhibitors on improving LVEDD but not on improving LVEDV. There is a need for much more research in the future due to the paucity of studies on SGLT-2 inhibitors and involved patients.

Of note, in 2018, a network meta-analysis involving 236 studies and 176,310 subjects found that GLP-1 agonists and SGLT-2 inhibitors were significantly associated with lower cardiovascular mortality than were the control treatments, and SGLT-2 inhibitors were associated with a reduction in heart failure events and myocardial infarction. This study is consistent with our results.

Although MET did not exert an effect on ventricular remodeling in our conclusion, a number of experimental and clinical studies have demonstrated that MET had a beneficial effect on lipids, atherosclerotic thrombosis, inflammation, endothelial function, oxidative stress, and antiproliferative and neuroprotective properties. Based on these findings, the recently published guidelines of the American Diabetes Association and the European Association for the Study of Diabetes recommended [74] either SGLT-2 inhibitors or GLP-1 agonists in patients with T2DM who are unable to achieve their target level of glycemic control with MET. Based on our results, MET treatment in combination with GLP-1 agonists or SGLT-2 inhibitors may also be a good choice for type 2 diabetic patients with cardiovascular disease.

Our results suggested that DPP-4 inhibitors exerted a significant negative impact on LVEDV. Zheng et al. [75] also found in their meta-analysis that the use of DPP-4 inhibitors was not associated with lower mortality than placebo or no treatment and that the use of SGLT-2 inhibitors or GLP-1 agonists was associated with lower mortality than DPP-4 inhibitors. Studies have found that DPP-4 inhibitor therapy did not increase the overall risk of major cardiovascular and renal outcomes but increased the hospitalization rate for heart failure [76]. Uncleaved brain natriuretic peptides, which are known to be substrates of the enzyme DPP-4, might be associated with decompensated HF [77]. Moreover, an increase in LVEDV should result in an increase in the ejection fraction according to the Frank-Starling law. However, our study found that although LVEDV increased with the use of DPP-4 inhibitors, LVEF did not increase. McMurray et al. found that vildagliptin had no major effect on LVEF but did lead to an increase in left ventricular volumes with type 2 diabetes and heart failure [38]. This finding was consistent with our results. A lack of EF increase suggests a negative impact of DPP-4 inhibitors on myocardial contractility. Studies have reported that DPP-4 inhibition is accompanied by increases in myocardial cAMP, which are related to potentiation of endogenous GLP-1. The increases in cAMP may exacerbate the clinical course of heart failure [78, 79]. DPP-4 inhibition might exacerbate the clinical course of heart failure via pathways of SDF-1 (stromal cell-derived factor 1)/CaMKII (Ca^2+^/calmodulin-dependent protein kinase II). These factors collectively might damage cardiomyocytes, which may be the reason why LVEDV increased, but LVEF did not [80]. Therefore, this result suggests that we should be cautious about the use of DPP-4 inhibitors in type 2 diabetic patients with cardiovascular disease because DPP-4 inhibitors may have adverse effects on cardiac function.

### Limitations

First, this paper included studies that covered three types of patients: one was type 2 diabetic patients, another was cardiovascular patients, and the third was patients with comorbidities of the first two. This may lead to between-studies heterogeneity and exert a certain impact on the combined results. Second, given that the sample size of the included studies ranged from 15 to 559, there was a lack of controlled clinical trials with a large sample size to conduct a more powerful demonstration of our outcome. Third, the variability of the ventricular structural changes estimated by echocardiography may exaggerate or ignore the therapeutic effect, leading to between-studies heterogeneity.

## Conclusions

As this network meta-analysis shows, GLP-1 agonists are more significantly associated with improved LVEF, LVESV and E/e′, SGLT-2 inhibitors are more significantly associated with improved LVEDD and E/e′, and DPP-4 inhibitors are more strongly associated with a negative impact on LVEDV than are placebos. SGLT-2 inhibitors are superior to other drugs in pairwise comparisons. Thus, GLP-1 agonist and SGLT-2 inhibitor treatment may serve as novel therapeutics for treating hyperglycemia and reducing cardiovascular comorbidities.

## Supplementary information

**Additional file 1: Figure S1.** Risk of bias graph with RevMan 5.3.

**Additional file 2: Figure S2.** Risk of bias summary with RevMan 5.3.

**Additional file 3: Table S1.** Treatment rankings.

**Additional file 4: Figure S3.** Funnel plot of mean difference of LVEF%.

## Data Availability

Not applicable.

## Abbreviations

CAD: coronary artery disease
CFR: coronary flow reserve
CI: confidence interval
CVD: cardiovascular disease
e′: early diastolic velocity
E/e′: mitral inflow E velocity to tissue Doppler e′ ratio
E/A: early diastolic to late diastolic velocities ratio
DPP-4: dipeptidyl peptidase-4
GLP-1: glucagon-like peptide-1
HF: heart failure
LVEDD: LV end-diastolic diameter
LVEDV: LV end-diastolic volume
LVEF: left ventricular ejection fraction
LVESD: LV end-systolic diameter
LVESV: LV end-systolic volume
LVMI: LV mass index
MD: mean difference
MET: metformin
RCT: randomized controlled trial
SGLT-2: sodium glucose cotransporter type 2
SU: sulfonylurea
T2DM: type 2 diabetes mellitus
TZDs: thiazolidinediones
STEMI: ST-segment elevation myocardial ischemia
SUCRA: surface under the cumulative ranking curve
