# Supplementary figures and images for: Effects of antidiabetic drugs on left ventricular function/dysfunction: a systematic review and network meta-analysis
Source: Cardiovasc Diabetol. 2020 Jan 22;19:10. doi: 10.1186/s12933-020-0987-x (PMC6977298; doi:10.1186/s12933-020-0987-x)

**Figure S1:** Risk of bias graph With RevMan 5.3

**
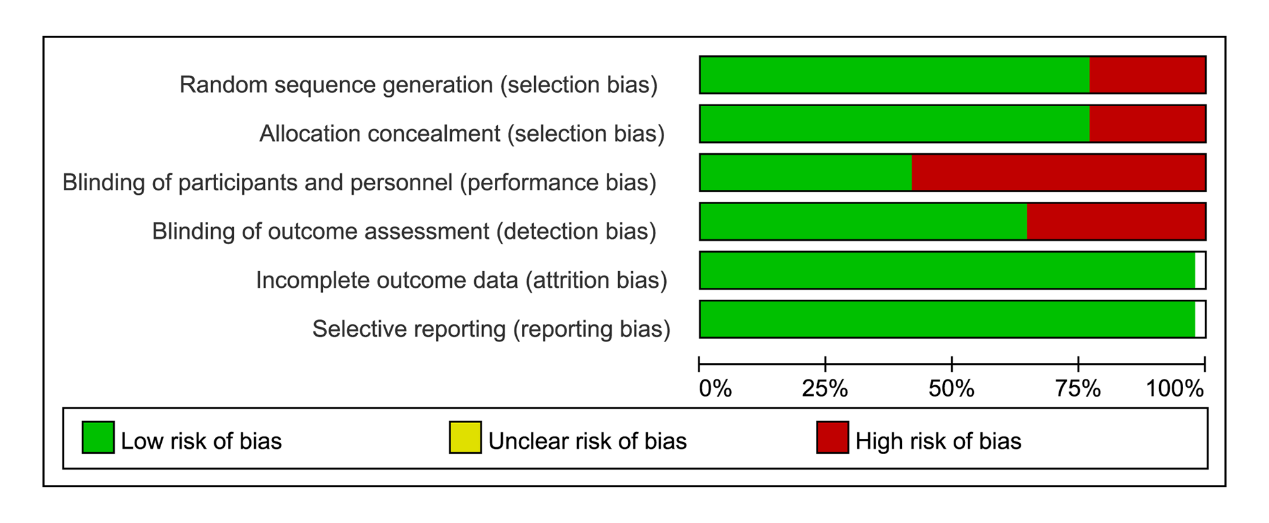
**

Supplement: Supplementary file 1 — Additional file 1: Figure S1. Risk of bias graph with RevMan 5.3. [file 12933_2020_987_MOESM1_ESM.docx]

**Figure S2:** Risk of bias summary With RevMan 5.3

**
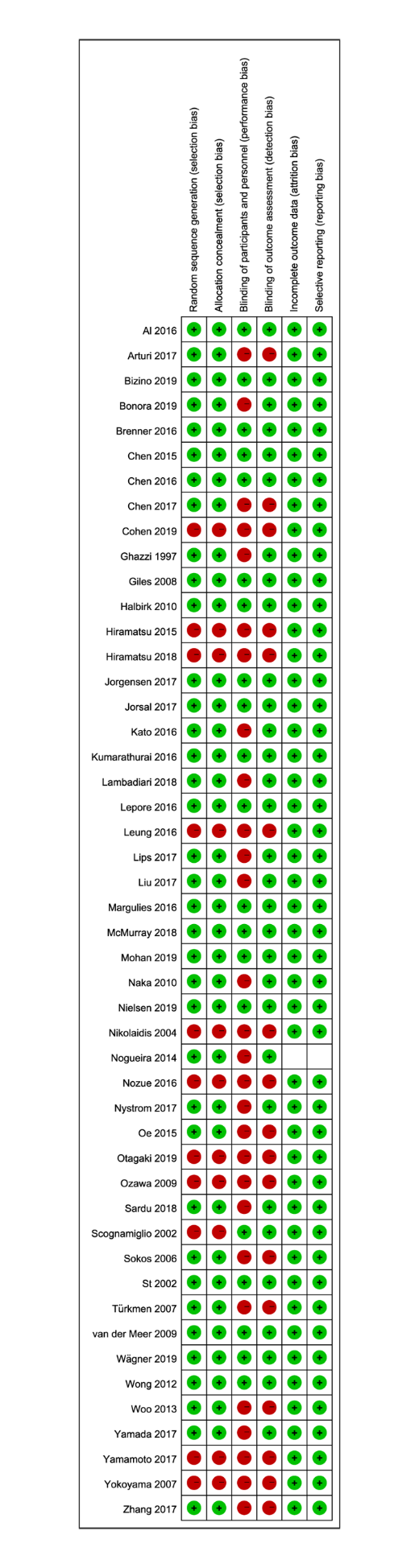
**

Supplement: Supplementary file 2 — Additional file 2: Figure S2. Risk of bias summary with RevMan 5.3. [file 12933_2020_987_MOESM2_ESM.docx]
