# Supplementary material for: Effects of antidiabetic drugs on left ventricular function/dysfunction: a systematic review and network meta-analysis
Source: Cardiovasc Diabetol. 2020 Jan 22;19:10. doi: 10.1186/s12933-020-0987-x (PMC6977298; doi:10.1186/s12933-020-0987-x)
Supplement: Supplementary file 3 — Additional file 3: Table S1. Treatment rankings. [file 12933_2020_987_MOESM3_ESM.docx]

**Table S1:** Treatment Rankings

| Treatment | LVESD | LVEDD | LVMI | LVESV | LVEDV | LVEF | e' | E/e' |
| --- | --- | --- | --- | --- | --- | --- | --- | --- |
| Placebo | 3 | 2 | 5 | 2 | 4 | 7 | 3 | 6 |
| MET | NA | 6 | 3 | 3 | 3 | 6 | 4 | 5 |
| GLP-1 agonist | 2 | 3 | 1 | 5 | 2 | 1 | 2 | 3 |
| DPP-4 inhibitor | 5 | 4 | 2 | 1 | 7 | 4 | 1 | 2 |
| SGLT-2 inhibitor | 1 | 1 | NA | NA | 1 | 5 | NA | 1 |
| TZDs | 4 | 5 | 6 | 4 | 6 | 2 | 5 | 7 |
| SU | NA | NA | 4 | NA | 5 | 3 | NA | 4 |

Note: **e’**: early diastolic velocity; **E/e’:** mitral inflow E velocity to tissue Doppler e’ ratio; **DPP-4**: Dipeptidyl Peptidase-4; **GLP-1**: Glucagon-Like Peptide-1; **LVEDD**: LV End-Diastolic Diameter; **LVEDV**: LV End-Diastolic Volume; **LVEF**: Left Ventricular Ejection Fraction; **LVESD**: LV End-Systolic Diameter; **LVESV**: LV End-Systolic Volume; **LVMI**: LV Mass Index; **MET**: Metformin; **SGLT-2**: Sodium Glucose Cotransporter Type 2; **SU**: Sulfonylurea; **TZDs**: Thiazolidinediones.
