# Supplementary material for: Effects of antidiabetic drugs on left ventricular function/dysfunction: a systematic review and network meta-analysis
Source: Cardiovasc Diabetol. 2020 Jan 22;19:10. doi: 10.1186/s12933-020-0987-x (PMC6977298; doi:10.1186/s12933-020-0987-x)
Supplement: Supplementary file 4 — Additional file 4: Figure S3. Funnel plot of mean difference of LVEF%. [file 12933_2020_987_MOESM4_ESM.docx]

**Figure S3:** Funnel plot of mean difference of LVEF%

**
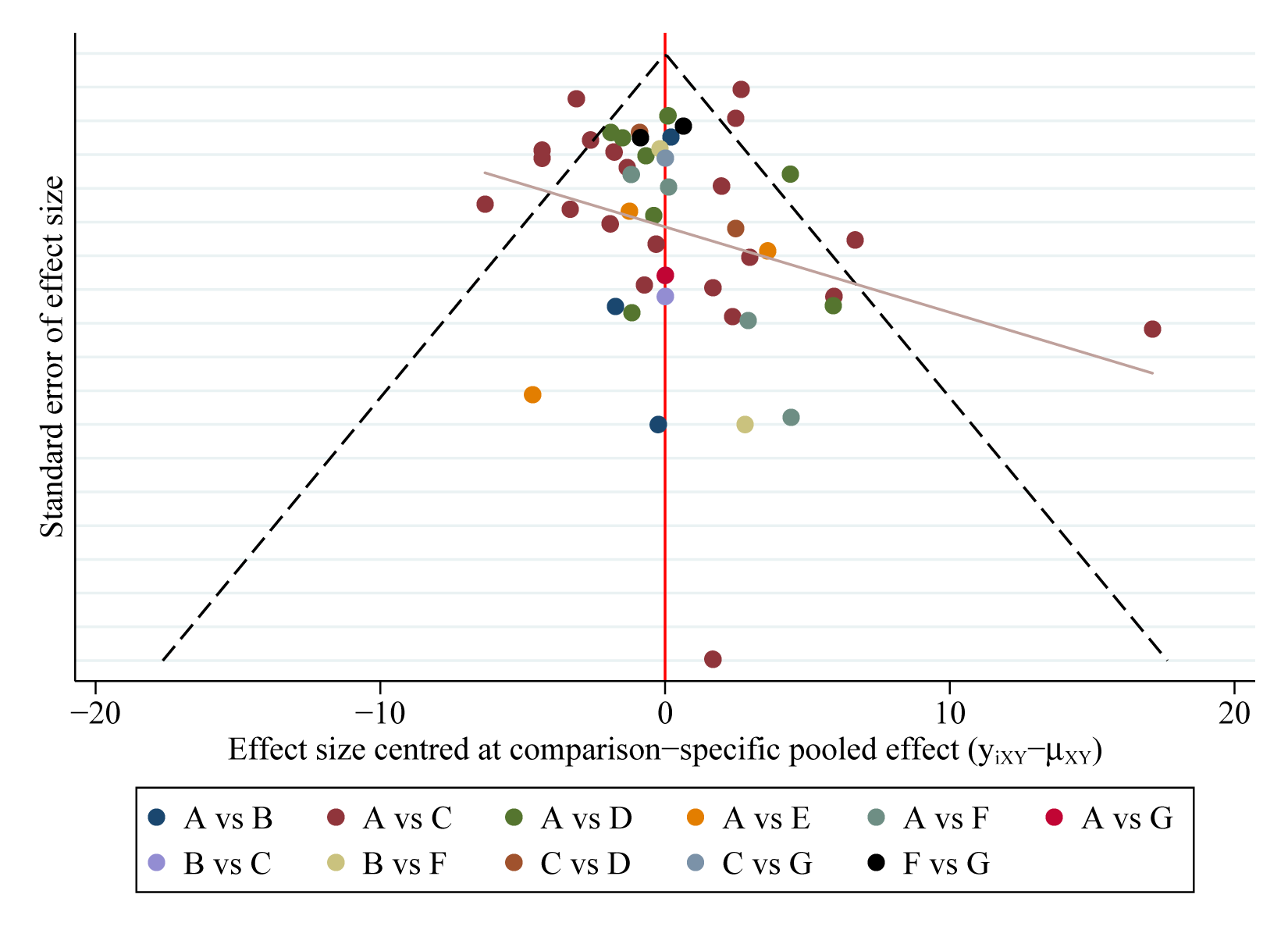
**

Note: A = Placebo, B = MET, C = GLP-1, D = DPP-4, E = SGLT2, F = TZDs, G= SU

**DPP-4**: Dipeptidyl Peptidase-4; **GLP-1**: Glucagon-Like Peptide-1; **MET**: Metformin; **SGLT-2**: Sodium Glucose Cotransporter Type 2; **SU**: Sulfonylurea; **TZDs**: Thiazolidinediones.
